# The Most Common Mistake in Laryngeal Pathology and How to Avoid it

**DOI:** 10.1007/s12105-020-01273-6

**Published:** 2021-03-15

**Authors:** Amin Heidarian, Bruce M. Wenig

**Affiliations:** Department of Pathology, Moffitt Cancer Center, Tampa, FL 33612 USA

**Keywords:** Spindle cell squamous carcinoma, Sarcomatoid carcinoma, Larynx, Upper aerodigestive traact, Sarcomas, Immunohistochemistry

## Abstract

Upper aerodigestive tract (UADT) spindle cell squamous carcinoma (SCSC), also known as sarcomatoid carcinoma, is a high-grade subtype of conventional squamous cell carcinoma (SCC) that is histologically characterized by a combination of differentiated SCC in the form of intraepithelial dysplasia and/or invasive differentiated SCC, and the presence of an invasive (submucosal) undifferentiated malignant spindle-shaped and pleomorphic (epithelioid) cell component. Typically, SCSC presents as a superficial polypoid mass not infrequently with surface ulceration precluding identification of an intraepithelial dysplasia. Further, in many cases an invasive differentiated SCC is not identified. Adding to the complexity in such cases, is that immunohistochemical staining in a significant minority of cases is negative for epithelial-related markers but often the cells express mesenchymal-related markers. In such cases, differentiating SCSC from a reactive (benign) spindle cell proliferation or a mucosal-based sarcoma can be problematic, with treatment implications. Herein, we detail the clinical and pathologic features of laryngeal SCSC and discuss the rationale for diagnosing a carcinoma and avoiding a diagnosis of sarcoma. In our experience, such cases represent one of the more common mistakes made in laryngeal pathology. Yet, virtually all such lesions are SCSCs. The treatment and prognosis relies on the accuracy of this distinction.

## Introduction

Squamous cell carcinoma (SCC) is the most common malignancy in head and neck pathology [1]. While conventional SCC accounts for the majority of cases, subtypes of SCC are seen in 10–15% of cases [2]. These subtypes include verrucous carcinoma, papillary SCC, spindle cell squamous carcinoma (SCSC), basaloid SCC, and adenosquamous carcinoma [1]. SCSC occurs throughout the upper aerodigestive tract mucosa (UADT) but is most common in the larynx. Classic histologic features are those of a biphasic malignant neoplasm to include differentiated SCC in the form of intraepithelial dysplasia and/or invasive differentiated SCC, and the presence of a malignant spindle-shaped and pleomorphic (epithelioid) cell component. In many cases, the biphasic malignant components are not present but rather may only include the malignant spindle-shaped and pleomorphic (epithelioid) cells (i.e., monophasic tumor). Considering the frequency of surface ulceration with absence of differentiated squamous cell component in up to one third of cases [3, 4], it can be challenging, especially in limited (biopsy) sampling to distinguish SCSC from a stromal (myo)fibroblastic proliferation or from a mesenchymal malignancy (i.e., sarcoma), even with the help of immunohistochemistry. While the spindle cells may be cytokeratin and/or p63 and p40 positive supporting the diagnosis of an epithelial malignancy [4, 5], in as high as 40% of cases cytokeratin staining is negative [4]; further, p63 and p40 may be negative, too [5]. Additionally, the spindle-shaped cells often express markers of purported mesenchymal cell derivation raising concern for the diagnosis of a sarcoma. Arguably, this differential diagnosis is among the more common laryngeal lesions surgical pathologists are confronted with. Too often the default diagnosis is a sarcoma in the absence of epithelial differentiation by light microscopy and immunohistochemical confirmation, and the presence of mesenchymal-related immunomarkers. As noted, such cases represent one of the more common mistakes that occur in laryngeal pathology.

## Clinical History

A 76-year-old man with no significant past medical history presented with a 6 month slow decline in voice quality. He has a limited remote smoking history stopping 45 years ago and does not drink alcohol. There are no additional symptoms such as dysphagia, odynophagia, ear pain or weight loss. Micro-laryngoscopy and biopsy were performed revealing a “substantial right vocal cord lesion” with infraglottic extension but no supraglottic extension. The biopsy diagnosis was high-grade leiomyosarcoma. Subsequently, a laryngectomy was performed revealing a 2.5 cm fleshy exophytic mass involving the right glottis and right true vocal fold.

## Histology

**Fig. 1:**
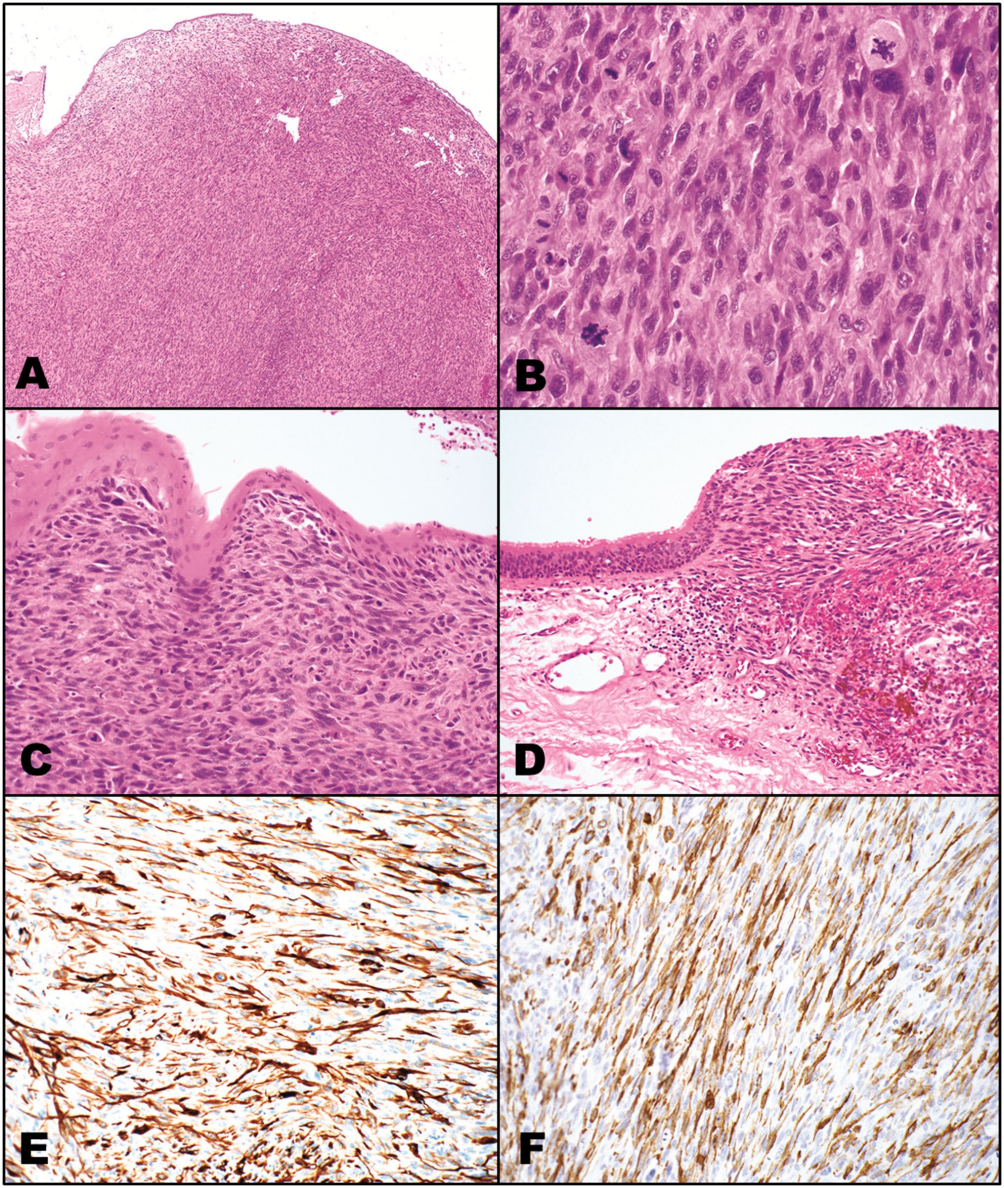
Spindle cell squamous carcinoma. **a** Polypoid neoplasm with intact attenuated surface squamous epithelium (top) exclusively comprised of a spindle-shaped cellular neoplasm with fascicular and storiform growth; **b** High-magnification shows the undifferentiated spindle-shaped and pleomorphic cells with marked nuclear pleomorphism and increased mitotic activity including atypical forms; **c** The malignant spindle-shaped cells are in direct continuity to the overlying intact squamous epithelium, the latter lacking dysplastic change; **d** Abrupt transition from intact benign ciliated respiratory epithelium to the malignant spindle-shaped cells with polypoid growth. The neoplastic cells are immunoreactive for **e** desmin and **f** smooth muscle actin. Of note, no immunoreactivity was present for epithelial markers (keratins, p63, p40) or for myogenin, MYOD1 and myoglobin

The laryngectomy specimen was sent in consultation. Microscopic examination showed a polypoid densely cellular undifferentiated spindle-shaped and epithelioid cell neoplastic proliferation with fascicular to storiform growth composed of cells with moderate to marked nuclear pleomorphism and increased mitotic activity, including atypical forms (Fig. 1a, b). There were scattered multinucleated tumor giant cells, as well as, osteoclastic-like multinucleated giant cells. While most of the immediately overlying surface was ulcerated, residual surface squamous and ciliated respiratory epithelium showed no evidence of intraepithelial high-grade dysplasia and there was no identifiable invasive conventional SCC component. However, in some areas, the malignant spindle-shaped cells were in direct contact with intact surface glottis squamous epithelium (Fig. 1c). In addition, areas showing abrupt transition from intact benign ciliated respiratory epithelium to the malignant spindle-shaped cells were identified (Fig. 1d). Non-descript vascularity was present and there was no association or merging of the neoplastic cells to blood vessels.

## Special Studies

Histochemical staining showed nonspecific trichrome staining without evidence of intracytoplasmic longitudinal striations. Immunohistochemical staining showed the neoplastic cells to be positive for desmin, muscle specific actin and smooth muscle actin (Fig. 1e, f) but negative for cytokeratins (AE1/AE3/CAM5.2 cocktail; OSCAR; CK5/6), p63, p40, myogenin, myoglobin, MyoD1, and S100 protein. Diffuse and strong p53 expression was present and a proliferation index rate of < 5% was seen by Ki67/CD45 staining. In situ hybridization was negative for Epstein-Barr encoded RNA (EBER).

## Discussion

SCSC occurs throughout the upper aerodigestive tract mucosa but is most common in the larynx (true vocal cords > > false vocal cords and supraglottis), followed by oral cavity (lips, tongue, gingiva, floor of mouth, buccal mucosa), skin, tonsil, and pharynx [1]. SCSCs account for about 2.7% of all benign and malignant laryngeal tumors and 4.2% of SCCs of larynx [6]. The majority of SCSC occur in men (85%) most frequently in 6th-8th decades of life [1, 2]. Symptoms are often present for a short duration owing to early clinical manifestations based on localization to the laryngeal glottis resulting in symptoms that include hoarseness, voice changes, airway obstruction, and dysphagia.

The development of SCSC has been linked to tobacco (cigarette smoking) and alcohol use [1]. SCSC has been reported in areas of prior irradiation following treatment for other mucosal-based carcinomas. Lewis [7] reported that a significant minority of patients with SCSC have a history of previous radiation to the originating site. Combining several large clinicopathologic studies of SCSC, 18% of the 326 cases occurred in a previously irradiated field at an average of 7 years and as late as 16 years later [7]. SCSCs are typically p16 negative and although SCSC rarely may harbor transcriptionally active high-risk human papillomavirus (HR HPV), the association has not been confirmed and does not appear to confer any prognostic benefit [8]. The treatment for SCSC is similar to that of conventional SCC usually necessitating surgical resection. Radiation therapy has not been felt to be effective in the treatment of SCSC. Overall prognosis is considered to be poor but early clinical stage disease (i.e., T1-T2) and/or polypoid glottis lesions have the most favorable prognosis compared to extraglottic and deeply infiltrating lesions. When SCSC metastasize, metastatic foci can be conventional SCC, sarcomatoid SCC, or a combination of both.

**Fig. 2:**
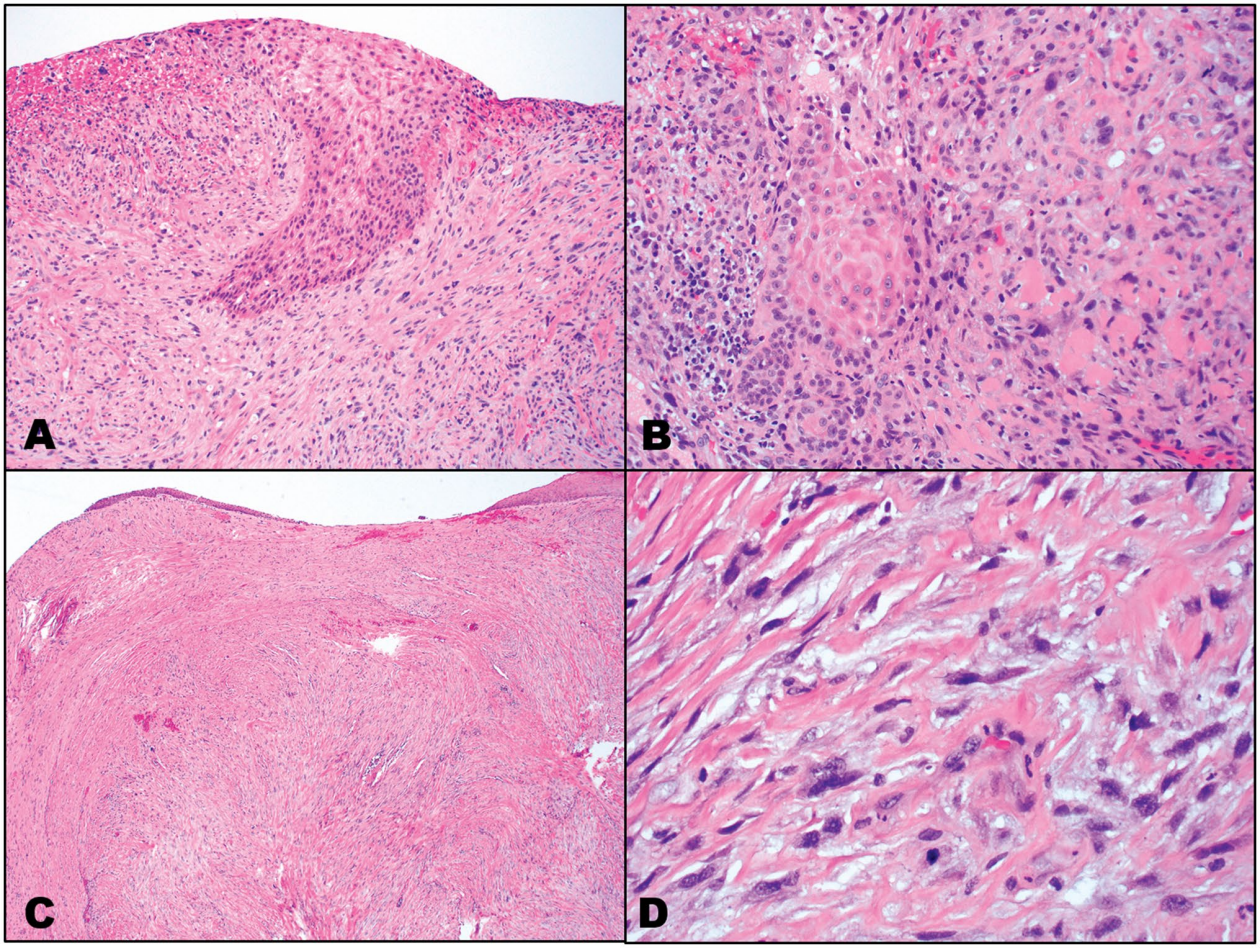
Spindle cell squamous carcinoma. Diagnostic biphasic cell components including: **a** high-grade intraepithelial dysplasia in association with the malignant spindle cells; and **b** Invasive keratinizing (differentiated) squamous cell carcinoma admixed with the malignant spindle cells. **c** Some cases are hypocellular and collagenized suggesting a nonneoplastic process at lower magnification; **d** At higher magnification there are areas with malignant cells including marked nuclear pleomorphism and hyperchromasia, and a mitotic figure (lower center)

UADT SCSCs, especially those of the larynx, are usually polypoid masses that are frequently ulcerated with associated necrosis. Histologically, in biphasic tumors there is a combination of differentiated SCC in the form of intraepithelial dysplasia and/or invasive differentiated SCC (Fig. 2a, b), and the presence of a malignant spindle-shaped and pleomorphic (epithelioid) cell component (Fig. 2a, b). However, in most cases the dominant cell type is the undifferentiated malignant spindle-shaped and pleomorphic (epithelioid) cells with growth patterns that may include fascicular, storiform, or palisading resembling sarcomas; hence, the designation sarcomatoid carcinoma. Typically, there is moderate to marked nuclear pleomorphism, increased mitotic activity including atypical mitoses, and tumor necrosis. Stromal collagenization is present and may vary from prominent to minimal, the former tending to be less cellular (pauci- to hypocellular) which may be referred to as collagenized (hypocellular) SCSC (Fig. 2c) [1]. Irrespective of the amount of stromal collagenization, the marked nuclear pleomorphism and hyperchromasia and increased mitotic activity that may include atypical forms are retained, allowing for a diagnosis of malignancy rather than a possible diagnosis of a reactive process (Fig. 2d). A myxoid stroma may occasionally be present varying from focal to more diffusely evident. Benign and malignant heterologous elements, including bone/osteosarcomatous differentiation, cartilage/chondrosarcomatous differentiation, and skeletal muscle/rhabdomyosarcomatous differentiation may be present in 7–15% of SCSCs [9, 10]. Despite the presence of heterologous elements, including malignant bone or cartilage, neither of these components have been reported to metastasize and in all probability represent a metaplastic phenomenon. Ansari-Lari et al. [3] reported the presence of identical p53 immunoreactivity in the epithelial and spindle cell components of SCSC, supporting the concept that these phenotypically divergent cell populations share similar developmental pathways and obviates the concept that SCSC represents a reactive process or a collision tumor between epithelial and mesenchymal components.

**Fig. 3:**
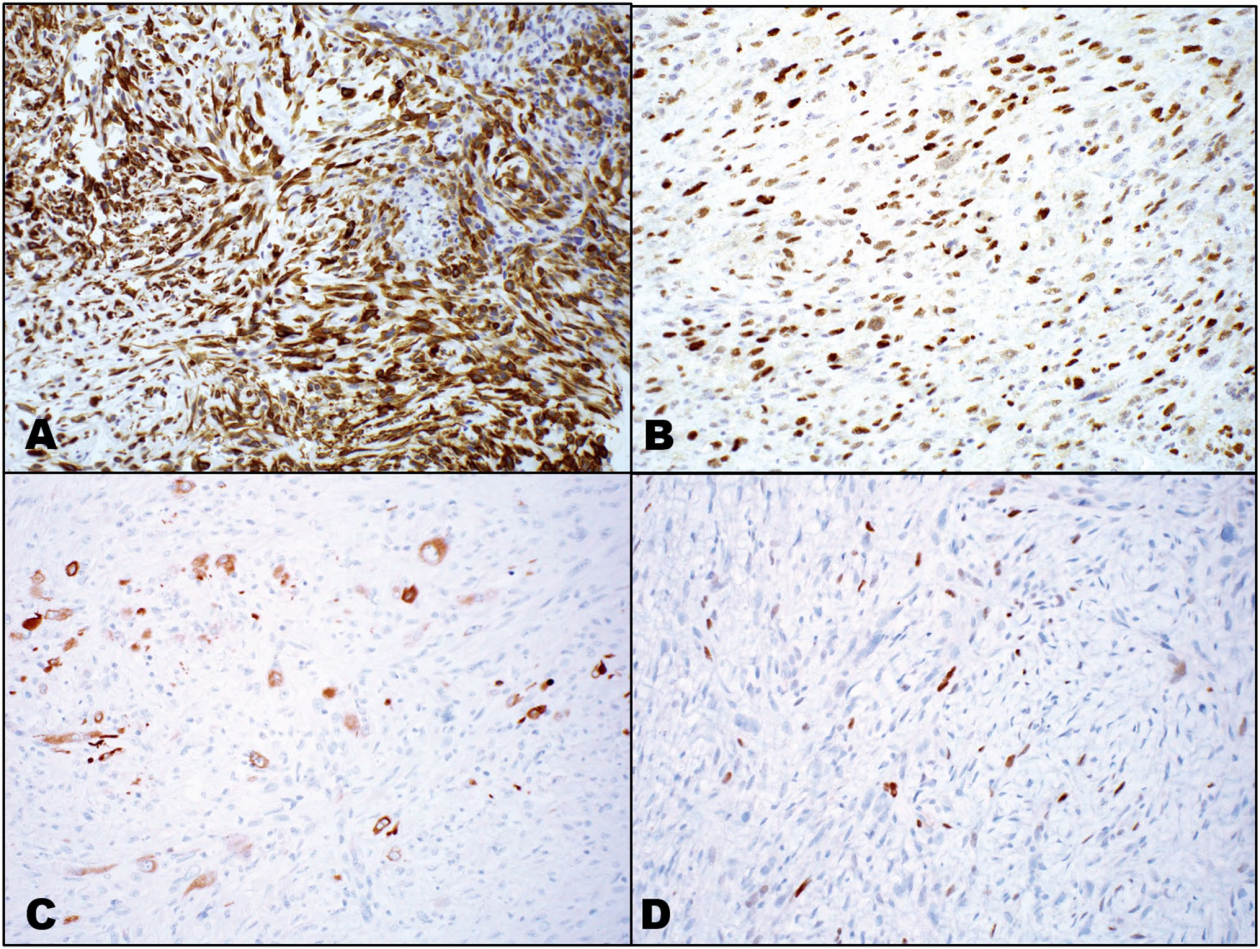
Spindle cell squamous carcinoma. Immunoreactivity can vary from case to case but in most cases there is evidence of epithelial differentiation with staining for cytokeratins and/or p63. Staining is variable, including: **a** diffuse and strong cytokeratin (AE1/AE3/CAM5.2 cocktail) and **b** p63. In other cases the extent of immunoreactivity is limited, including: **c** focal cytokeratin (AE1/AE3/CAM5.2 cocktail) and/or **d** patchy p63 reactivity

Histochemical staining in SCSC is of limited utility. The spindle cells are cytokeratin and/or p40/p63 immunoreactive in the majority of cases [1, 4, 5] (Fig. 3a). Broad spectrum cytokeratin staining should be used and often necessitates using multiple keratins (e.g., AE1/AE3, CAM5.2, OSCAR, CK5/6) in order to identify reactivity. Keratins and/or p63 and p40 reactivity may vary from focal to diffuse. In as high as 40% of cases, cytokeratin staining is negative [4]; further, p63 and p40 may be negative, too. Nuclear p63 and/or p40 immunoreactivity often mirrors cytokeratin reactivity but may be positive in cases lacking cytokeratin staining [5]. Of note, p63 staining is more consistently positive than p40 [5], but even so may be very limited and detected only in scattered malignant cells [1]. The absence of cytokeratin and/or p63 and p40 staining does not exclude a diagnosis of SCSC. Vimentin reactivity is consistently identified (100% of cases) and tends to be diffuse and strong. Various myogenic markers, including desmin and actins may be present [1]. Coexpression of mesenchymal markers and epithelial markers (i.e., cytokeratins) may occur. In the absence of rhabdomyoblastic differentiation, other myogenic markers including myogenin and myoglobin typically are not present. p16 staining is usually negative but p16 positivity may be present in a minority of cases and p16-positive cases tend to be located in the oropharynx [7].

SCSC harbors complex genetic alterations. Choi et al., [11] in a study on SCSC, documented concordant loss of heterozygosity or retention of heterozygosity in 80% of cases supporting the contentions that sarcomatoid carcinoma evolves from the conventional epithelial-type carcinoma, affirming the malignant nature of the sarcomatoid component, and indicating that there is molecular progression associated with the sarcomatoid transformation.

A definitive diagnosis of UADT SCSC can be rendered in the presence of a superficial (polypoid or exophytic) neoplasm showing biphasic features including conventional squamous cell carcinoma including intraepithelial dysplasia and/or invasive differentiated SCC associated with submucosal malignant spindle-shaped and pleomorphic (epithelioid) cells. Challenging diagnostic issues arise in spindle cell lesions devoid of squamous differentiation by light microscopy, absence of immunoreactivity for epithelial-related markers and expression of markers correlating with presumptive mesenchymal cell origin including vimentin, actins and myogenic markers, thereby, resulting in an erroneous diagnosis of a sarcoma.

The question posed is why are such malignancies not a sarcoma? This is a valid question and the following is an approach to assist in avoiding such a mistake. Overall, primary sarcomas of the larynx are rare, representing 0.3–1% of all laryngeal malignancies [6]. The most common laryngeal sarcoma is chondrosarcoma; other types of laryngeal sarcomas may occur including (but not limited) to rhabdomyosarcoma, leiomyosarcoma and synovial sarcoma. In general, non-matrix producing sarcomas of the UADT, including the larynx, are extremely rare, tend to be deeply-seated in any given location, and do not usually result in a polypoid mass protruding from a mucosal surface as typically occurs in SCSC. As a rule, a malignant spindle cell neoplasm of a mucosal surface of the UADT presenting as an exophytic polypoid lesion or identified in the more superficial locations of the submucosa should be considered as SCSC. This is true even in the absence of a differentiated squamous epithelial component and/or the absence of immunoreactivity for epithelial markers. As stated in the 2017 World Health Organization of Classification of Head and Neck Tumors, “true sarcomas of the larynx/hypopharynx are rare, and a malignant spindle cell neoplasm arising in these sites is best considered an spindle cell squamous cell carcinoma until proven otherwise” [12].

As previously noted, heterologous elements including rhabdomyoblastic differentiation may be present in SCSC [6] as well as in other types of head and neck malignant neoplasms [10]. Primary laryngeal rhabdomyosarcomas (RMS) are rare, and when they occur tend to be a pediatric tumor and typically are embryonal subtype. Laryngeal RMS is rare in adults greater than 30 years of age, in contrast to SCSC which predominate in the much older aged patients [6]. Laryngeal RMS in adults tends to be alveolar subtype. Of note, alveolar RMSs frequently express epithelial as well as neuroendocrine markers which could complicate their diagnosis especially in head and neck sites where it may be mistaken for SCC or a neuroendocrine carcinoma [9, 13]. In general, alveolar RMS will harbor recurrent fusion transcripts (*PAX3-FOXO1* and *PAX7-FOXO1* fusions) in 80% of cases. As such, if a diagnosis of adult RMS is in question, molecular analysis would assist in diagnosing RMS.

Another myoid type malignancy to be considered would be a leiomyosarcoma (LMS). UADT LMSs are rare owing to the relative absence of smooth muscle in mucosal sites. The most common site for UADT mucosal-based LMS is the sinonasal tract, taking origin from smooth muscle of vascular structures, and may occur as a post-radiation sarcoma [14]. Laryngeal LMS is an exceedingly rare tumor with less than 10 reported cases in the English literature [15]. UADT LMSs are characterized by the presence of intersecting fascicles of elongated cells with cigar-shaped blunt-ended nuclei and eosinophilic fibrillary cytoplasm, and intimate relationship and merging of the tumor cells with blood vessels walls. Such features are not present in SCSC. Epstein-Barr virus (EBV)-associated smooth muscle tumors occur in immunocompromised patients as a complication of renal transplantation and immunosuppression, acquired immunodeficiency (AIDS), and cardiac and/or liver transplantation [16]. Most EBV-associated smooth muscle tumors occur in children with a tendency to develop in organs not typically common sites for LMS including liver, lung, heart, colon, soft tissue, spleen, and dura [16, 17]. To the best of our ability we could not identify any literature reports of EBV-associated LMS of the UADT.

As previously noted some examples of SCSC may be hypocellular and collagenized suggesting a reactive process such as granulation tissue or even a myofibroblastic-dominant lesion such as nodular fasciitis or inflammatory myofibroblastic tumor (IMT), the latter reported occurring in the larynx [18]. IMTs are moderately cellular with a proliferation of spindle-shaped cells including enlarged nuclei but with ample basophilic appearing cytoplasm (low nuclear-to-cytoplasmic ratio) and an absence of a striking degree of nuclear pleomorphism. Intranuclear eosinophilic inclusions may be identified. Inflammation shows a predominance of lymphocytes, plasma cells and eosinophils. Mitotic figures may be encountered but atypical mitoses are not seen. The findings of atypical mitoses should prompt consideration of a malignancy. IMTs are not encapsulated but they do not exhibit the insidious pattern of infiltration of adjacent tissues which is characteristic of malignant neoplasms such as SCSC. IMTs typically lack staining for epithelial markers (e.g., cytokeratins, p63, and p40) although focal cytokeratin reactivity may be seen. In addition, the myofibroblastic cell component may be muscle specific actin (HHF35), smooth muscle actin, and vimentin positive. IMTs may show the presence of anaplastic lymphoma kinase (ALK) gene rearrangements and its expression in IMT is indicative of oncogenic ALK expression representing an important mechanism in the pathogenesis of IMT and supporting the concept that IMTs are neoplastic. Of note, while > 90% of pediatric IMTs show gene rearrangements, 90% of fusion negative IMTs occur in adults [19], so the absence of ALK would not exclude a possible diagnosis of IMT. ALK-negative IMTs have alterations of *ROS1* and *PDGFRB*, among other genes [17]. ROS1 antibody can be utilized as a reliable predictor of *ROS1* gene rearrangement [20]. Additionally, evidence of *ETV6-NTRK3* fusion has been reported in ALK-negative IMTs [21].

Rarely, mucosal malignant melanoma may occur in the larynx [22] and include epithelioid and/or spindle-shaped cells. However, melanomas will be immunoreactive for S100 protein and SOX10, as well as melanoma-related markers including HMB45, Melan A, tyrosinase, MART1 and MITF1, findings that will be absent in carcinomas or sarcomas.

## Conclusions

Differentiating upper aerodigestive tract SCSC from sarcoma can be challenging, especially with limited tissue sampling. SCSCs are superficial mucosal lesions, an uncommon feature with sarcomas. In the presence of biphasic histologic features, including differentiated SCC (i.e., intraepithelial dysplasia and/or invasive differentiated SCC) coupled with the presence of a malignant spindle-shaped and pleomorphic cell component, a diagnosis of SCSC can be rendered. Challenges occur when a given neoplasm only manifests the spindle-shaped cell component precluding a definitive diagnosis by light microscopy alone. In such instances, the identification by immunohistochemistry of epithelial differentiation using cytokeratins, p63 and p40 can support the diagnosis. However, in a significant minority of cases, epithelial markers will be lacking. Adding to the complexity in these cases is that many SCSCs express markers associated with mesenchymal cell origin including vimentin and myogenic markers (e.g., desmin, actins). As detailed in this manuscript, in spite of lacking all evidence of epithelial differentiation, such superficially situated malignancies invariably are SCSC and should be managed accordingly, and should not be mistaken for a sarcoma.
